# The host range of generalist and specialist phages in capsule-diverse *Klebsiella* hosts is driven by the evolvability of receptor-binding proteins

**DOI:** 10.1371/journal.pbio.3003515

**Published:** 2025-11-26

**Authors:** Celia Ferriol-González, Pilar Domingo-Calap

**Affiliations:** Institute for Integrative Systems Biology, University of Valencia-CSIC, Paterna, Spain; Universitat zu Koln, GERMANY

## Abstract

Capsule diversity is a major limiting factor for phage host range in capsulated bacterial hosts. Phage receptor-binding proteins (RBPs) recognize the capsule and initiate infection, making them key players in phage tropism. In this study, we applied an experimental evolution approach to investigate host range adaptation in a diverse 12-phage community interacting with a *Klebsiella* spp. community containing 39 distinct capsular types. Our findings revealed that generalist phages possessed highly evolvable RBPs, accumulating non-synonymous mutations that modulated their host range. In contrast, specialist phages acquired fewer mutations but remained stable in the community, maintaining their narrow host range. Additionally, recombination between co-infecting closely related phages facilitated rapid host range adaptation through RBP swapping. However, most recombined genes encoded endonucleases or proteins of unknown function, suggesting their potential role in phage survival. This study advances our understanding of phage host range evolution and provides new insights for optimizing phage-based applications.

## Abstract 2

Capsule diversity limits phage host range by affecting RBP interactions in capsulated bacteria. This study shows that generalist phages evolve host range through RBP mutations and recombination, while specialist phages remain stable, offering insights into phage adaptation and strategies for enhancing phage-based therapeutic applications.

## Introduction

Phages, viruses that infect bacteria, are ubiquitously distributed in nature, playing a crucial role in shaping microbial communities and driving ecosystem dynamics [1,2]. Their interactions with bacterial hosts influence nutrient cycling, microbial evolution, and human health. A critical aspect of phage ecology is the host range, defined as the diversity of bacterial hosts that a phage can infect [3]. Elucidating the determinants of phage host range is critical, as it reveals the specificity and adaptability of phages while illuminating the coevolutionary dynamics between phages and bacteria. This understanding is indispensable for applications such as phage therapy and biotechnology, where manipulating the phage host range can yield significant benefits. Host range is a wide continuum, but overall, phages can be categorized as specialists, which exhibit narrow host ranges—often limited to a single or a few strains—and generalists, which can infect multiple strains or even different bacterial species [3]. In nature, phages continually modify their host range through coevolutionary processes with their hosts, counter-adapting to resistance mechanisms to overcome phage infection [1]. Although current isolation techniques tend to underrepresent generalist phages, recent metagenomic studies indicate that broad-range phages may be more prevalent than previously thought [1,3,4]. Previous studies described that the evolutionary success of broad host range phages is influenced by ecological factors such as host diversity and density [3,5,6]. In host-diverse environments, generalist phages can persist even when susceptible hosts are scarce, though they often incur fitness costs that reduce their virulence in individual hosts. Consequently, host diversity may favor the selection of lower-fitness generalists within viral populations [5].

The host range is primarily determined by the recognition and attachment process, which is mediated by phage receptor-binding proteins (RBPs) that specifically interact with bacterial receptors typically exposed on the cell surface [7]. In some cases, successful infection requires multiple RBP-host receptor interactions [8]. Bacteria often develop resistance to phages by modifying, hiding, or downregulating these receptors, thereby evading detection [9–11]. In response, phages can adapt by altering their RBPs through amino acid substitutions and conformational changes. This capacity to undergo adaptive evolution to acquire new functions, in this case, targeting different receptors, is known as evolvability [12,13]. Moreover, phages can also acquire complete or partial RBPs from other phages via horizontal gene transfer, ultimately modifying their host range [14,15]. In capsulated bacteria, the exopolysaccharide capsule provides protection against diverse environmental stresses, serves as a key virulence factor, and acts as the primary barrier to phage infection [16–18]. To overcome this barrier, many phages have evolved specialized RBPs and depolymerase enzymes that specifically recognize and degrade the oligosaccharide components of the capsule [19,20]. Given the high diversity of bacterial capsules [17], these structures have been proven to be the main determinants of phage host tropism [21,22]. Particularly, *Klebsiella* spp. has over 180 distinct capsular types (K-types) identified to date [23], and most *Klebsiella* phages are highly specific, typically infecting only one or two K-types [22,23]. In addition, after recognition, adhesion, and phage entry, additional bacterial defense systems may disrupt the infection cycle by degrading phage nucleic acids, interfering with DNA replication or transcription, or avoiding phage propagation by aborting infection [24–29]. In response, phages have developed diverse mechanisms to counteract these defenses. The increased availability of phage and bacterial genomic data has accelerated the discovery of these bacterial defenses and phage anti-defenses, although many remain uncharacterized or undiscovered [30].

Here, we aimed to investigate how phages targeting capsulated bacteria evolve and adapt within a capsule-diverse bacterial community. We hypothesized that phage RBPs, as the primary determinants of tropism toward capsulated hosts, would undergo modifications that enable phages to infect new bacterial strains. Using *Klebsiella* spp. as a model, we implemented an experimental evolution framework to track phage adaptation in a genetically and phenotypically diverse bacterial community. This approach revealed the critical role of RBP diversification, including changes mediated by horizontal gene transfer, providing valuable insights into the evolutionary mechanisms that drive phage host range expansion.

## Results

### Implementing an experimental design to study phage community evolution

**Fig 1:**
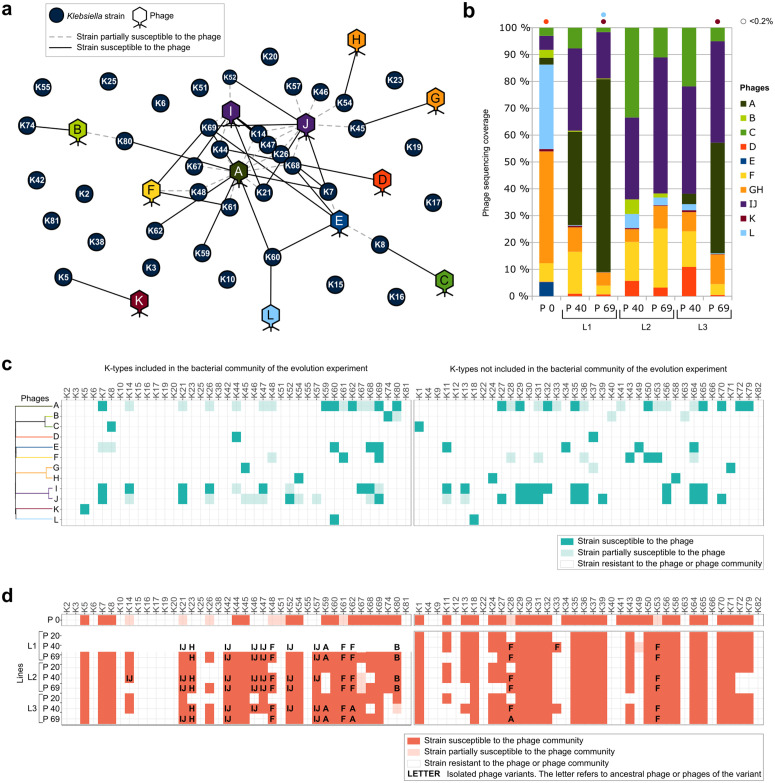
The host range and the composition of the phage community evolved during the experiment. **a.** Phage–strain interactions network at passage 0 based on the host range of the phages of the initial community. **b.** Evolution of the composition of the phage community in terms of sequencing coverage. Phages at proportions lower than 0.2% are represented as dots. The data underlying this Figure can be found in S1 Data. **c**. Genomic distances between phages in the community are provided in the phylogenetic tree. The matrix consists of the host range of each phage in the capsular types of the 77 *Klebsiella* reference strains collection, with both strains included and not included in the bacterial population. The data underlying this Figure can be found in S2 Data. **d.** Evolution of the host range of the phage community at different passages of the three lines. Letters represent the ancestors or parental phages of the variant isolated in each passage-strain combination. P: passage, L: line. The data underlying this Figure can be found in S3 Data.

An in-house collection of *Klebsiella* phages was used to investigate how phage communities adapt to host-diverse environments [23]. Among them, 12 double-stranded DNA (dsDNA) lytic phages (*Caudoviricetes*) were selected to construct a phage community for the experimental evolution (Table 1). This community comprised phages from eight families and nine different genera, exhibiting broad phylogenetic diversity with minimal intergenomic similarity except among closely related taxa. The host range of each phage, both individually and in combination (10⁸ plaque-forming units [PFUs]/mL per phage), was evaluated via serial dilution spot tests against the 77 *Klebsiella* reference serotypes from the Statens Serum Institut (Denmark) (S1 Table), revealing marked differences in host specificity (Fig 1A). A subset of 39 *Klebsiella* strains from this reference collection was selected for the bacterial community, where 15 strains were susceptible to the phage community, 18 were fully resistant, and 6 displayed intermediate susceptibility. At passage 0, we inoculated the initial phage community (10^6^ PFUs per phage) into the bacterial community (10⁷ colony-forming units [CFUs]) in three independent evolution lines. Following a 4-h incubation, bacteria were removed by centrifugation to isolate the phage fraction, ensuring that the bacterial community did not evolve during the experiment. The phage community was subjected to 69 passages of experimental evolution. During even-numbered passages, the phage titer was assessed to confirm that the inoculum was maintained and that the phage community was retained throughout the experiment.

**Table 1:** Description of the initial phage community. The table includes each phage letter, phage name, K-type of its isolation strain, host range classification (G: generalist, S: specialist), taxonomic information, and source. A phage is classified as a specialist if it infects only a single bacterial strain within the community used in the evolution experiment, and a generalist if it can infect multiple strains.

Phage	Name	Isolation strain (K-type)	Number of strains at passage 0		Host range	Genome length	Family / subfamily	Genus	Source	ENA sample accession
			Susceptible	Partially susceptible						
A	vB_Kte_K65PH164	K65	6	9	G	166,435	*Straboviridae*	*Jiaodavirus*	Ferriol-González and colleagues, 2024	SAMEA114518338
B	vB_Ko_K74PH129C2	K74	1	1	S	41,794	*Autographiviridae*	*Przondovirus*	Ferriol-González and colleagues, 2024	SAMEA114518346
C	vB_Kpl_K8PH128	K8	1	0	S	40,536	*Autographiviridae*	*Przondovirus*	Ferriol-González and colleagues, 2024	SAMEA114518359
D	vB_Kpl_K44PH129C1	K44	1	0	S	43,860	*Autographiviridae*	*Vectrevirus*	Ferriol-González and colleagues, 2024	SAMEA114518364
E	vB_Kpn_K34PH164	K34	3	2	G	48,306	Unclassified	Unclassified	Ferriol-González and colleagues, 2024	SAMEA114518392
F	vB_Kpn_K50PH164C1	K50	2	1	G	48,936	*Drexlerviridae*	Unclassified	Ferriol-González and colleagues, 2024	SAMEA114518388
G	vB_Kpn_K45PH128C2	K45	1	0	S	145,364	*Vequintavirinae*	*Mydovirus*	Ferriol-González and colleagues, 2024	SAMEA114518342
H	vB_Kpl_K54lambda1.1.1	K54	1	0	S	143,319	*Vequintavirinae*	*Mydovirus*	Ferriol-González and colleagues, 2024	SAMEA114518340
I	vB_Kpn_K30lambda2.2	K30	7	3	G	111,917	*Demerecviridae*	*Sugarlandvirus*	Concha-Eloko and colleagues, 2023	SAMEA114518077
J	vB_Kpn_K7PH164C4	K7	4	8	G	113,079	*Demerecviridae*	*Sugarlandvirus*	Concha-Eloko and colleagues, 2023	SAMEA114518078
K	vB_Ko_K5lambda5	K5	1	0	S	160,642	*Ackermannviridae*	*Taipeivirus*	Ferriol-González and colleagues, 2024	SAMEA114518361
L	vB_Kpn_K60PH164C1	K60	1	0	S	73,440	*Schitoviridae*	*Gamaleyavirus*	Ferriol-González and colleagues, 2024	SAMEA114518337

### Host diversity shapes the composition of the phage community by favoring generalist phages

Relative abundances of phages within the community were expected to fluctuate throughout the experiment. To monitor these changes, we sequenced the phage community at passages 0 (initial), 40 (phage community with broader host range), and 69 (end of the experimental evolution) in each evolution line, and we calculated the relative abundance of each phage (Fig 1B). Our results revealed that the community was consistently dominated by generalist phages, particularly the *Sugarlandviruses* (phages I and J) and phage A in lines 1 and 3. In contrast, specialist phages B, K, L, and *Mydovirus* (G and H) declined markedly over time, even though they persisted at lower levels, while specialist phages C and D increased in abundance, especially when phage A was absent (line 2) or present at lower amounts (passage 40 of line 3). Overall, 9 of the 12 phages were maintained until the end of the experiment across all three lines, with 2 phages (A and B) lost in one line and 1 phage lost in all lines (phage E). The occasional loss of a phage in a single line likely reflected the inherent stochastic variability of the experimental system. Notably, phage E was eradicated in all three lines, indicating a reproducible pattern in phage–bacteria interactions that drives its extinction. Although phage E efficiently infected three primary hosts within the bacterial community, the intense competition for these hosts may result in the loss of the phage. On host K60, for example, phage E competed with phage L (which is highly specific to K60 and potentially more efficient) and generalist phage A. Similarly, for hosts K68 and K69, phage E was outcompeted by generalist phages I and J, and, in the case of K69, also by phage F. The ability of these competing phages to exploit alternative hosts likely enhances their persistence in the community, ultimately disadvantaging phage E.

### Phages that predominantly adapt their host range are generalists

To explore the adaptation of the phage community to the bacteria, we analyzed the infectivity of the phage combination against the 39 *Klebsiella* strains included in the experiment (Fig 1C and 1D). We assessed phage infectivity at passages 20, 40, and 69 across the three evolution lines by serial dilution spots and compared the results with those of the initial phage community. Over time, the number of susceptible strains increased, with the broadest host range observed in line 1 at passage 40. The phage community expanded its infectivity to 10 strains that were initially fully or partially resistant in at least one passage of one line. Conversely, infectivity decreased in 8 strains initially susceptible either fully or partially. This may be attributed to fluctuations in phage titers or the loss of certain phages throughout the experiment. To further validate how phages adapted to the experimental conditions, we evaluated the host range of the evolved communities against the 38 *Klebsiella* strains from the reference collection that were not part of the experimental bacterial community. Infectivity decreased in 10 of these 38 strains in at least one passage of one line, while only 3 strains exhibited improved infectivity. Notably, adaptation of phage communities followed a consistent pattern: infectivity acquired against initially resistant strains was often accompanied by a loss of infectivity against others. Moreover, changes in infectivity rarely occurred in only one line, and when they did, they correlated with the line-specific loss of a particular phage from the community. For a deeper study of the host range modifications, we isolated, purified, and sequenced individual plaques from passages 40 to 69 across the three lines. Plaques were isolated from 13 hosts where phage infectivity improved in at least one passage of one line, and 4 hosts were already susceptible to the initial phage community.

Of the 96 combinations tested, 87 phage plaques were successfully sequenced (S2 Table). These isolated phages were variants of 6 of the original community, exhibiting different mutations, including recombination events. By sequence homology, we identified the ancestral phages from which these variants were derived. Among the 13 strains where infectivity improved, we isolated variants of the *Sugarlandviruses* (phages I and J, *n* = 27) in 6 strains, phage F (*n* = 38) in 5 strains, phage A (*n* = 10) in 1 strain, and phage H (*n* = 6) in 1 strain. Except phage H, all these ancestral phages efficiently infected multiple *Klebsiella* K-types within the experimental bacterial community, indicating that generalist phages were the primary drivers of host range changes in the viral community. Interestingly, the host K80 was susceptible to the initial phage community due to its sensitivity to phage A. However, plaques recovered from this host were variants of phage B (*n* = 6), a specialist phage to which K80 was initially only partially susceptible. This suggests that mutations in these variants enhanced the ability of phage B to infect this host, indicating a potential evolutionary shift in host range adaptation.

### The mutational landscape of evolving phages differs between specialists and generalists

**Fig 2:**
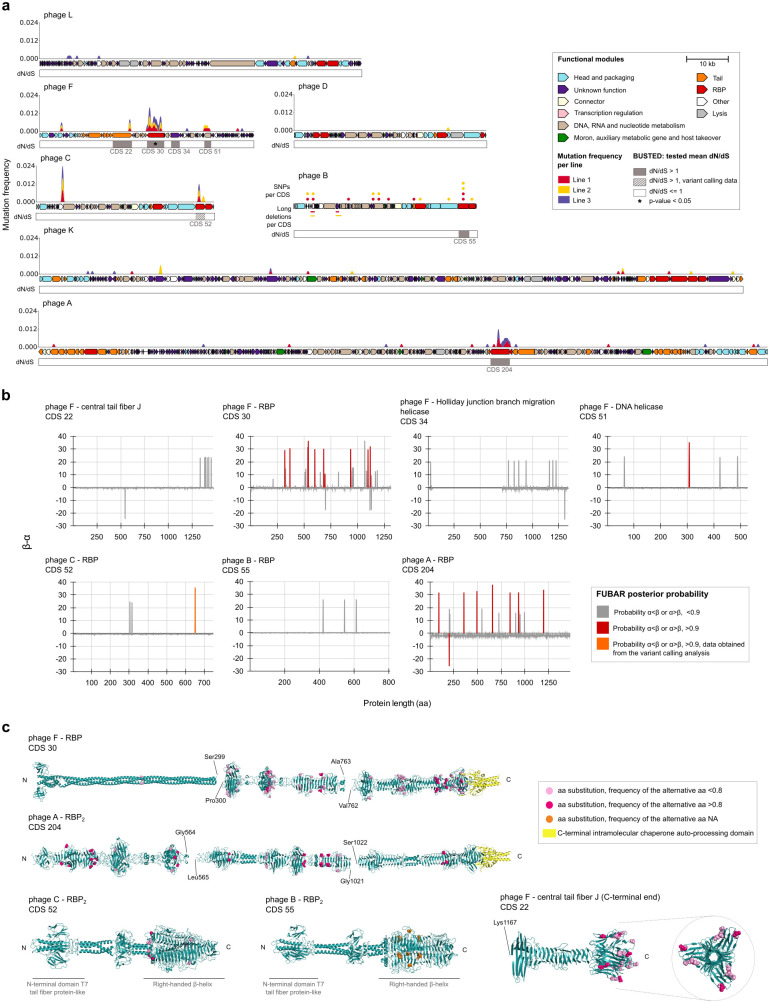
Genomic annotation, variability accumulation, and analysis of proteins under positive selection of phages that did not recombine during the experiment. **a.** First, the graph represents the mutation frequency considering mutations detected in sliding windows of 500 bp in the variant calling data for phages A, C, D, F, K, and L. The representation of the annotated genomes was performed using the R package gggenomes [75]. Due to technical reasons, data presented for phage C are variable positions and long deletions per coding sequence (CDS) detected in the whole genome alignment of its isolated variants. Dots represent single-nucleotide polymorphisms, and lines represent deletions. Colors of curves, dots, and lines correspond to the evolution line where mutations were detected. The CDSs with dN/dS ratios >1 and *p*-values < 0.05 are indicated in the lower bar. The data underlying this Figure can be found in S4 Data. **b.** Graphic representation of the *β*–*α* value for each position of the CDS, with dN/dS > 1 indicating exact positions under positive selection. Bayes factor for the prediction of positive selection on positions with posterior probabilities of [*α* < *β*] <0.9% were >10 (strong evidence). aa: amino acid. The data underlying this Figure can be found in S6 Data. **c.** 3D folding of the recognition proteins affected by mutations in the different lines. Variable positions detected in the protein sequence alignment of the variants were indicated as spheres. Frequencies >0.8 of the alternative amino acids were indicated in hot pink when the data were available in the variant calling analysis. The data underlying this Figure can be found in S7 Data.

Given the phenotypic changes observed in the evolving phage communities, we investigated the genomic variability that emerged during the experiment. We performed variant-calling analyses to identify single-nucleotide polymorphisms, insertions, and deletions (indels) using sequencing data from passages 0, 40, and 69 across all three evolution lines. Due to the high intergenomic similarity among phages within the same genus, this analysis was limited to phages lacking closely related counterparts in the initial community at similar frequencies. When possible, we complemented this with alignment-based analyses of the isolated variants to further characterize the genetic modifications (Fig 2A). Two distinct mutational profiles emerged. In phages D, K, and L, mutations were infrequent, dispersed throughout the genome, and did not show consistent patterns across different lines, suggesting these mutations were likely neutral or noncritical for adaptation. In contrast, phages A, B, C, and F displayed a more structured mutational landscape, with mutations accumulating in specific proteins across multiple lines where these phages persisted. Phages A and F showed a strong accumulation of mutations within RBPs, confirmed by a significant enrichment compared to the rest of the genome (Comparison of Poisson rates test, phage A, *p*-value < 1 × 10^−11^; phage F, *p*-value < 1 × 10^−6^, S3 Table). This parallelism across lines suggests strong selective pressure on these regions. Phages B and C displayed fewer RBP mutations, mostly shared between lines, with lower substitution rates compared to generalists (Comparison of Poisson rates test, phage B, *p*-value < 0.05; phage C, *p*-value ~ 0.1, S3 Table). In phage C, mutations also clustered in a serine-threonine kinase gene (*p*-value < 1 × 10^−6^, S3 Table), forming a haplotype possibly linked to host adaptation through the modification of a single domain of this protein. Additional parallel deletions were detected in phage B variants, which were associated with enhanced phage amplification (ANOVA, Tukey’s multiple comparisons of means, *p*-value = 4.8 × 10^−7^, S1 Fig and S5 Data).

### Diversifying selection targets RBPs in generalist phages

To assess whether the mutated proteins were driven by selective pressure, we analyzed the ratio of non-synonymous to synonymous substitutions (dN/dS) using the Branch-Site Unrestricted Statistical Test for Episodic Diversification (BUSTED) [31]. This test was applied to nucleotide sequences of proteins from evolved variants isolated via plaque assays, which are representative members of the evolved phage communities, most of them isolated on hosts where the phage improved their infectivity. When direct variant sequences were unavailable, we alternatively used consensus sequences derived from variant-calling data across different evolutionary lines (Fig 1A and S4 Data). Seven proteins exhibited a dN/dS ratio greater than 1: four RBPs, one central tail fiber J, and two helicases. However, only the RBP of phage F yielded a statistically significant *p*-value (< 0.05), indicating strong evidence of this protein being under positive or diversifying selection. To further investigate selection at the amino acid level, we applied the FUBAR test (Fast, Unconstrained Bayesian AppRoximation for Inferring Selection) [32] to the proteins with dN/dS > 1. Unlike BUSTED, FUBAR identifies individual amino acid sites under selection by estimating the difference between non-synonymous (*β*) and synonymous (*ɑ*) substitution rates, assigning a posterior probability to each position to determine whether *ɑ* < *β*. The RBP of phage F and the RBP_2_ of phage A had the highest numbers of sites with a posterior probability > 0.9 and a Bayes factor > 10, providing strong evidence of diversifying selection acting on these regions (Fig 2B). These findings are consistent with host range shifts observed in the plaque-isolation of variants.

### Protein folding and mutation distribution differ between RBPs of specialist and generalist phages

To explore the spatial distribution of mutations, we predicted the 3D structures of all RBPs that accumulated mutations during evolution using AlphaFold3 [33], annotated their functional domains via InterProScan [34], and mapped the variable residues (Fig 2C). Notably, the most mutated RBPs (RBP of phage F and RBP_2_ of phage A), shared structural features. Both exhibited an elongated architecture with a C-terminal intramolecular chaperone autoprocessing domain, typical of tail fiber proteins [23]. In both cases, mutations were primarily located in the loop regions of the β-helix domains, frequently involved in host recognition and receptor binding [35–37]. In contrast, RBP_2_ from phages B and C, from which we detected limited or no host range expansion, adopted a structurally distinct and more rigid tail spike fold, characterized by a C-terminal right-handed β-helix with depolymerase activity. Notably, no variants of phage C were recovered by plaque isolation, and all phage B variants were isolated exclusively from K80, a strain partially susceptible to the ancestral phage. This suggests that this structural rigidity may constrain their evolutionary adaptability to novel *Klebsiella* capsular types. Additionally, in phage F, we identified a significant accumulation of amino acid substitutions (posterior probability *ɑ* < *β* > 0.85; Bayes factor ~8) at the C-terminal end of the central tail fiber J protein. This region has been associated with evasion of the Tai antiphage defense system, suggesting that some of the observed mutations may contribute to immune escape [38].

### RBPs of generalist phages exhibit increased evolvability

The high frequency of mutations observed in the RBPs of phages A and F and the evidence of positive selection suggested that these RBPs were more prone to adaptive evolution than other genomic regions. This mutational accessibility, combined with the generalist phenotype and enhanced infectivity on novel hosts, indicated that these RBPs had higher evolvability. In this context, we understand evolvability as the RBP’s ability to modify host infectivity (phenotype). To validate whether RBP mutations resulted in phenotypic shifts, we first assessed the correlation between variant calling and whole-genome alignment across phages A and F (S2A Fig). This analysis confirmed that mutations at sites under positive selection in plaque-isolated variants also arose in the phage community. To address detection bias and technical thresholds due to the low number of variants isolated in some line/passage combinations, we compared the detection of positively selected mutations (allele frequency > 0.2) across both methods. Summing all line/passage combinations evaluated, for phage F, a positively selected position was identified by both approaches 10 times, 3 times only by whole-genome alignment, and 1 time exclusively by variant calling (Fisher’s exact test *p*-value = 0.596). Similarly, for phage A, 16 times these positions were detected by both methods, 3 times uniquely in the alignment, and 1 time only in the variant calling (Fisher’s exact test *p*-value = 1.0). Interestingly, most mutations detected in the alignments were present at low frequencies, consistent with their occurrence in individual variants.

After that, we selected seven isolated variants of phage F and four of phage A, each carrying different combinations of mutations with high allele frequency (≥ 0.8) and/or strong positive selection (Bayes factor > 10) for phenotypic characterization. All selected variants were isolated at passage 40, which is the one that exhibited the broadest host range across all the evolution lines. Each variant carried additional background mutations (S4 Table). We assessed the efficacy of plating (EOP) related to the ancestral phage and its isolated variants on strains susceptible or partially susceptible to the ancestral phage or any of its variants, independently of its inclusion in the bacterial community employed in the evolution experiment (S2B Fig). Regarding changes in the EOP in the new isolation hosts of the evolved variants, we detected a notable increase in cases where the isolation host was initially resistant or poorly susceptible to the ancestral phage (EOP < 0.02). In contrast, when the ancestral phage already exhibited moderate to high infectivity on the isolation host (EOP > 0.2), the EOP of the evolved variant was generally maintained rather than increased. EOP differences in other strains diverged depending on the variant. Phage F variants showed more pronounced changes than phage A, likely due to their narrower initial host range. Most phage F variants improved infectivity (≥ 1.7 log_10_(EOP) increase) in strains where the ancestral phage was poorly effective, while variants from already susceptible strains showed minimal changes. EOP differences in variants of phage A were much less pronounced. For both phages, some variants also had modified infectivity in strains not included in the evolution experiment, suggesting broader adaptation. In rare cases, infectivity increased drastically without shared mutations, indicating possible non-genetic mechanisms, such as host anti-defense systems.

### RBP-swapping between co-infecting closely related phages alters host range

**Fig 3:**
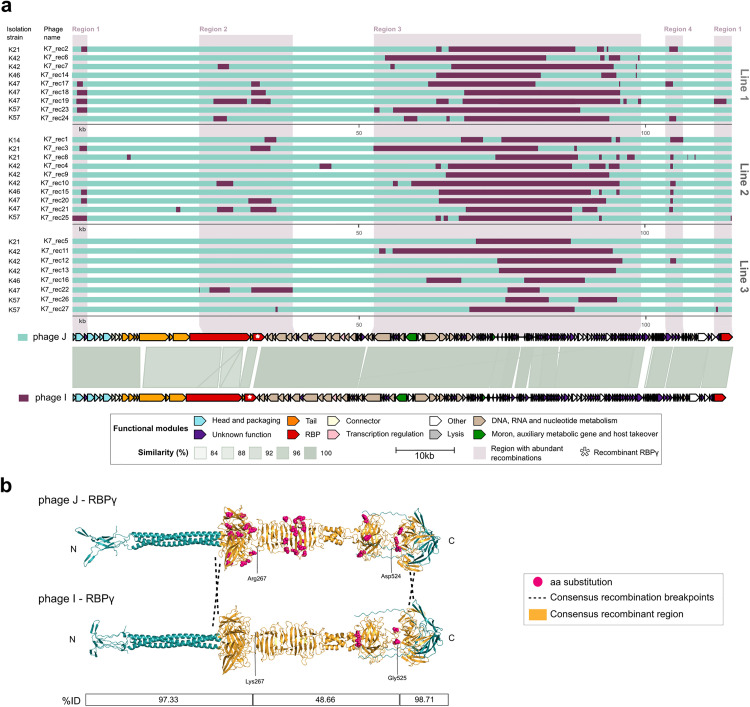
Graphic representation of the recombination events in the *Sugarlandvirus* I and J variants. **a.** Representation of the variants isolates in the different strains in the three lines. Genome fragments of each parent are represented in a different color. Regions with the most abundant recombination events are marked and associated with a schematic representation of the functional annotation and intergenomic similarity between the two parental phages. **b.** 3D folding of the RBPγ. The consensus recombinant region, which is associated with the infection of the K-type 47, is represented in light orange. Variable positions detected in the protein sequence alignment of the variants are represented as hot pink spheres. The table indicates the percentage of identity of the parental protein sequences in three protein regions limited by the amino acids indicated. ID: identity between protein sequences.

**Fig 4:**
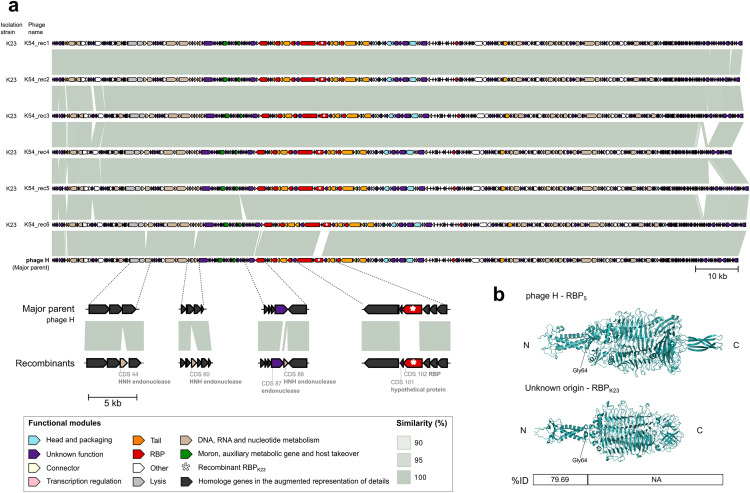
Genome alignment of the variants and ancestral *Mydovirus* H evidences its recombination with a sequence of unknown origin. **a.** Functional annotation and intergenomic similarity of the 6 variants isolated in K-type 23 and ancestral phage H. The representation of the annotated genomes was performed using the R package gggenomes [75]. A detailed view of the unknown origin genes acquired by all the isolated variants is provided. **b.** 3D folding of the ancestral RBP_5_ and acquired RBP_K23_ that swapped during the experiment. RBP_K23_ is associated with the acquisition of infectivity in K23. The table indicates the percentage of identity of the parental protein sequences. ID: identity between protein sequences, NA: no alignment.

Genome assemblies of the isolated phage plaques revealed that recombination contributed to the evolution of several phage lines. In particular, the closely related *Sugarlandviruses* I and J, which share 90.3% intergenomic similarity, could co-infect some host strains during the experiment. We identified 27 recombinant variants between these two phages, all with phage J as the predominant genomic background (major parental, Fig 3A). To assess the functional impact of these recombination events, we focused on the three RBPs of phages I and J: RBPɑ, RBPβ, and RBPγ [39]. Notably, recombination affecting a fragment of RBPγ was strongly associated with the ability to infect *Klebsiella* strains carrying the K-type K47. Among the three RBPs, RBPγ showed the greatest sequence divergence between the parental phages (78.92% amino acid identity), with most differences concentrated in the central region (Fig 3B). All recombinants isolated from K47 strains acquired this divergent region of the RBPγ from phage I, suggesting a key role in host specificity. Furthermore, sequence alignments revealed the presence of diverse substitutions in RBPγ, particularly in phage J, probably due to the highest number of variants isolated encoding this RBP (20/27). This is a pattern of diversification similar to the one observed in RBPs in other generalist phages, such as A and F. Beyond coinfection-driven recombination between lytic phages, we also observed recombination events involving prophages. Some host strains harbored prophages that became active during the experiment (S5 Table), contributing to phage diversification. For instance, all phages isolated from strain K23 were variants of phage H that acquired a complete RBP (RBP_K23_) through recombination, likely from a prophage (Fig 4). RBP_K23_ closely matched tail spike proteins from other *Mydoviruses* infecting K-type K23 from *Klebsiella* (vB_Kpn_K23PH08C2, KpS8, and vB_KpnM_Seu62; > 97.4% similarity), suggesting its key role in host specificity [23,40].

### Endonucleases and hypothetical genes are predominantly acquired through recombination

Detailed alignment and recombination analysis of the 27 recombinants between *Sugarlandvirus* I and J revealed that most recombination events clustered in a genomic region rich in genes of unknown function, nucleic acid metabolism, morons, auxiliary metabolic genes, and involved in host takeover (Fig 3A, region 3). These features are often implicated in interactions with bacterial defense systems. These characteristics suggest that this region may represent a genomic hotspot for adaptive evolution and phage-bacteria arms races. However, no known anti-defense systems were detected using Anti-DefenseFinder [29,41,42]. Interestingly, despite the high genomic similarity between the two parental phages, only one gene exclusive to the minor parent (phage I) was consistently retained in all isolated recombinants. This gene, annotated as coding sequence (CDS) 98, encodes a hypothetical protein containing a GIY-YIG endonuclease domain, an enzymatic motif commonly involved in DNA cleavage and associated with selfish genetic elements and phage invasion mechanisms [43]. A similar trend was observed in *Mydovirus* H recombinants isolated from K23 strains (Fig 4A). In addition to acquiring a novel RBP (RBP_K23_), these variants incorporated several other genomic elements not present in the parental phage, including three genes encoding endonucleases, a partial gene fragment, and a complete hypothetical protein. None of these sequences was shared with the coevolving *Mydovirus* phage G, pointing toward recombination with a cryptic prophage during the experiment. Altogether, these findings suggest that recombination facilitates host range shifts via RBP exchange but also enables the acquisition of diverse functional genes that may enhance phage fitness by modulating host interactions, defense evasion, or genome packaging.

## Discussion

This study explores the evolution of phage host range in a diverse and stable *Klebsiella* spp. community, where the variability of the bacterial capsule presents the principal barrier to phage infection. Our findings confirm that selective pressures in environments with high host diversity drive the accumulation of mutations in some RBPs. Although it has been demonstrated that amino acid substitutions can drastically alter host specificity [5,44,45], our data validate that the evolvability of the RBPs is a determinant of host-range modifications in capsule-diverse hosts. In an ecological context, our experimental model sheds light on the strategies that allow phages to coexist and adapt within complex microbial communities. Previous work focused on studying the evolution of a single or a few phages in host-diverse settings, revealing competitive dynamics between generalists and specialists [5]. By contrast, our study began with a heterogeneous dsDNA phage community, and it shows that while generalist phages tend to predominate over successive passages, specialist phages can persist if their specific hosts remain available. This balance underscores the importance of inter-phage competition in structuring the community and shaping survival strategies [46,47]. Notably, a phage (E) that adopted both roles depending on its competitors for different hosts was consistently outcompeted, suggesting that the associated fitness trade-offs of each role ultimately compromised its survival. Indeed, the being specialist or generalist appears to be context-dependent, modulated by the availability of hosts and the competitive landscape [6,48–50].

In our work, generalist phages demonstrated modifications in host infectivity, correlating with parallel accumulation of mutations in their RBPs, which has also been observed in studies with *Salmonella*, *Pseudomona*s, or *Escherichia* *coli* phages [5,51,52]. It is worth discussing that generalist phages, by definition, have theoretical access to a higher number of hosts, which could result in more mutations. However, having access to a broader diversity of hosts does not necessarily correlate with the amount of replication due to generalists’ fitness trade-offs [5,53]. Our experimental design is limited by not considering other central factors in this aspect, like burst size, latent period, or potential nonproductive adsorption, which restricts our conclusions about whether the replication of generalists is more extensive than for specialists [54]. However, the number of variable positions observed per phage does not correlate with the number of initially susceptible hosts, being, for example, phage F the phage with the most mutated RBP, and phage A the most generalist.

The isolated variants of generalist phages showed differences in infectivity (measured through changes in EOP) compared to the parentals. Each variant had its adaptation pattern, either improving or decreasing infectivity depending on the host, rather than sharing a universal generalist phenotype. Additionally, host range modifications were much more pronounced in phages with more new susceptible hosts (phage F). Other sources of variability may be considered when evaluating the host range difference between variants. Each variant contains its pattern of high-frequency positively selected mutations and other mutations exclusive to each one, creating each variant’s unique mutational background. In addition, epigenetic variability may also contribute to phenotypes observed [30]. All these prevent us from knowing the exact effect of each single adaptive mutation.

Structural analyses of generalist phages’ RBPs revealed that these proteins typically adopt tail fiber-like architectures, which are known to confer broader host ranges in *Klebsiella* phages [23]. These architectures appear inherently more adaptable, favoring evolutionary plasticity. In contrast, specialist phages exhibited limited RBP diversification. Mutations in these proteins were generally restricted to a few conserved residues, and the RBPs maintained a classical tail spike conformation characterized by a right-handed β-helix depolymerase domain—an architecture associated with high structural stability and narrow host range [22,23,39,55]. The rigidity of this structure likely constrains evolutionary change, as disruptive mutations are less tolerated given the stability requirements for proper folding [13,35,37]. Thus, a key insight from our study is the relationship between structural stability and evolvability. RBPs with high structural stability, such as those with right-handed β-helix domains, are less able to accommodate adaptive changes without compromising protein folding. Conversely, tail fiber-like RBPs, which exhibit greater conformational plasticity and require chaperone assistance for assembly, are predisposed to adaptive mutations that enable host-range shifts [13,56–58]. This conformational flexibility is essential for high evolvability, potentially allowing these RBPs to explore alternative structures that may facilitate the recognition of novel host receptors [13,59]. In some instances, conformational rearrangements alone were sufficient to alter receptor specificity [60], emphasizing the central role of protein architecture in directing evolutionary pathways.

Recombination events emerged as significant drivers of host-range evolution in our system. Phages that acquired an RBP by swapping and consequently gained access to novel hosts were preferentially enriched when isolating phage variants in these new hosts, as exemplified by recombinants of *Sugarlandvirus* and *Mydovirus*. This enrichment suggests that recombination can be a rapid evolutionary mechanism to overcome host barriers, complementing point mutations in the adaptation process. Notably, the recombination events were not confined to RBP-coding regions. In *Sugarlandvirus*, frequent exchange of broader genomic segments enriched in genes of unknown function was observed. This not only underscores the vast expanses of “genomic dark matter” within phage genomes [61] but also raises the possibility that these uncharacterized genes might be key for roles such as antibacterial defense, modulation of host interactions, or the regulation of phage-phage competition. Moreover, recent studies are challenging the traditional view of homing endonucleases as merely selfish genetic elements with an invasive nature [43,62]. Evidence now suggests that these elements may also interfere with productive infection by related, co-infecting phages, thereby influencing overall infection dynamics and phage community structure. Our findings correlate with this emerging perspective by suggesting that these poorly understood genomic regions, including noncanonical regulatory elements, can affect phage survival and host-range adaptation.

Our study provides new mechanistic insights into the constraints and drivers of phage host-range evolution. The divergent evolvability of generalist and specialist phages has broad implications for understanding phage-host coevolution in dynamic environments, including the human microbiota. In addition, these findings are particularly relevant for developing phage-based therapeutic strategies. By elucidating the molecular and structural determinants of host-range adaptation, our work informs the design of phage applications that could be more robust and effective in targeting bacterial pathogens. Future work using RBP engineering, such as structure-guided mutagenesis or RBP swapping, could further validate and extend these findings and decipher the targets of highly evolvable RBPs in capsular bacteria, ultimately contributing to refined approaches to phage therapy and microbial ecology.

## Materials and methods

### Bacterial strains

The *Klebsiella* reference strains collection, corresponding to the 77 *Klebsiella* reference serotypes, was purchased from the Statens Serum Institut (Copenhagen, Denmark). The genomes of 62 of the 77 reference strains were available online (S1 Table). The collection includes diverse *Klebsiella* species: *K. pneumoniae*, *K. planticola*, *K. oxytoca*, *K. ozaenae*, and *K. terrigena*. All bacteria were grown in Luria–Bertani (LB) with CaCl_2_ (3.78 mM) broth at 37°C at 180 rpm.

### Bacterial community preparation for experimental evolution

A bacterial pool encompassing 39 reference strains of 4 different *Klebsiella* species (39 different K-types) was created for the experimental evolution as a model for a host-diverse environment (S1 Table). An exponential culture of each bacterial strain was prepared at an OD_620_ = 0.2 (~10^8^ CFU/mL). All cultures were mixed in the same proportion to elaborate the stock of the bacterial community for the passages. The combined culture was concentrated and stored at −70°C in aliquots with 20% glycerol for its preservation.

### Phage community preparation

The 12-phage community evolved in the experiment was previously designed and tested in a previous work [23]. This community combined large dsDNA phages, with a continuum of host ranges and genomic diversity, including representatives of 9 different genera (2 *Sugarlandvirus*, 2 *Przondovirus*, 2 *Mydovirus*, 1 *Vectrevirus*, 1 *Taipeivirus*, 1 *Jiaodavirus*, 1 *Drexlerviridae* unclassified, and 1 unclassified family) (Table 1). For the elaboration of the community, phages were propagated separately in a final volume of 5 mL LB + CaCl_2_ broth using each isolation strain. They were finally combined to a final 10^8^ PFU/mL titer per phage. Reads and assemblies of the ancestral phages were already available (Table 1). Genomes were reassembled using Unicycler (version 0.5.0) [63] and annotated using Pharokka (v1.4.1) [64] (S4A Data). Specific identification of RBPs was performed by protein alignment with the already available annotation of the ancestral genomes performed in previous work, where RBP annotation was specifically refined [23,39].

### Experimental evolution of the phage community in a host-diverse environment

A bacterial pool aliquot was resuspended in LB (OD_620_ = 0.2). For the first inoculum (passage 0), 10^8^ PFU/mL per phage of the initial community were added to the bacterial pool in three 2 mL tubes in a final titer of 10^6^ PFU/mL per phage, initiating three evolution lines. Tubes for each line were incubated for 3.5 h at 37°C at 750 rpm. After that, they were centrifuged to eliminate bacteria (passage 1), and the supernatant was diluted to adjust the inoculum for the next passage. To maintain an adjusted titer during the experiment, every 2 passages, the phage community was titrated in at least 3 strains. A total of 69 passages were performed for the three independent lines.

### Host range evaluation

For the initial phage community, the host range was first tested for passage 0 (10^8^ PFU/mL per phage) to detect possible phage-host interactions not reported under the conditions assayed in our previous work [23]. In addition, the host range was evaluated throughout the experimental evolution in passages 20, 40, and 69. Serial dilutions for each line (and passage) were tested for the phage communities using the spot test technique (dilutions from 1 to 10^−6^) in the 77 reference collection as described before [23]. An interaction was considered positive only if single plaques were consistently observed in the serial dilutions in the different replicates. Turbid spots only present in the 10^−1^ dilution were considered ambiguous interactions. The absence of a visual spot was considered a negative interaction. Two replicates of the experiment were performed, and three for the doubtful cases.

### Single-plaque isolation of phages with an adapted host range

Plaque isolation was performed in newly adapted hosts and 4 already susceptible hosts. A dilution of the passage corresponding to the limiting dilution observed in the host range evaluation for each strain was plated, a total of 96 strain-passage combinations. When plaques were observed, they were recovered and purified as described before [22]. After phage purification, they were propagated in their isolation strain and concentrated using the Concentrating Pipette Select System (Innovaprep) with pipettes Ultra (<0.05 µm). Titration of selected variants of phages A and F and ancestors to compare their EOP in a subset of strains was performed as described previously for host range evaluation. EOP in each strain was calculated as (titer in the evaluated strain)/(titer in the isolation strain of the ancestor). When no spot was detected for a phage-bacteria combination, a 10^2^ PFU/mL concentration was considered, as a value under the detection limit for the technique that allowed further calculations. The EOP considered was the average of the two replicates performed. Differences in EOP were calculated as follows: Log_10_(EOP_variant_)−Log_10_(EOP_ancestor_).

### Viral genome sequencing

Passages 0, 40, and 69 were sequenced from the phage communities as single plaques. The removal of host DNA and the digestion of phage capsids were performed as described before [22]. Extraction and purification of DNA were done using DNA Clean and Concentrator 5-Kit (Zymo) for the passages and using Maxwell PureFood GMO and Authentication Kit (Promega) with Maxwell RSC Instrument (Promega) for single plaques. Sequencing libraries were prepared using the Illumina Nextera XT DNA kit (paired-end reads 2 × 250 bp). Reads were generated in the Illumina MiSeq platform with MiSeq Reagent Kit v2 for the evolution passages and MiSeq Reagent Kit v2 nano for the single plaques. Sequencing read quality was assessed using FastQC software (version 0.11.9, Babraham Bioinformatics) [65]. Sequencing data from the passages is available in the BioProject PRJNA1164145. De novo genome assembly for the passages was carried out with the “metaspades” function of SPAdes (version 3.15.4) [66,67]. For genomic data from single plaques, Unicycler (version 0.5.0) [61] in combination with SPAdes (version 3.15.4) [66] was used. If necessary, the assembly was done with a subset of 10,000 reads and refined with Pilon (version 1.24) [68]. Sequences of single phage variants are available in GenBank (Accession: PQ569649-PQ569735) (S2 Table). Ancestral phage genomes were also reassembled under this pipeline (Table 1). Read mapping for coverage calculation was performed using BBMap [69].

### Genomic characterization of the phage communities

The intergenomic similarity and genomic distance between phages were assessed using VIRIDIC [70]. To avoid nonspecific read mapping, only phages with an intergenomic similarity lower than 2 with any other phage in the community were considered sufficiently different to be analyzed by variant calling. For this reason, small regions with a high percentage of identity (higher than 80%) were excluded from the analysis. For phages with higher intergenomic similarities but sufficient difference in sequencing depth (ratio of the percentage of reads per position lower than 1:4), variant calling analysis was also performed for the phage with the highest sequencing depth. Reads were mapped using BWA (version 0.7.17) [71], and the variants were called using LoFreq (version 2.1.5) [72]. In parallel, read mapping was visualized using Integrative Genomics Viewer (IGV) [73]. CDS with mutations were translated using MEGAX [74] to check whether the mutations were synonyms or non-synonyms and whether there were amino acid changes. For visualization of mutations detected along the genome of the ancestral phage, the mutation frequency was calculated on sliding windows of 500 bp for each line. A graphic representation of this value was overlaid with the graphic representation of ancestral phage annotation obtained with the R package gggenomes [75].

To analyze the possible activation of prophages from the bacterial community, unmapped reads were assembled using the “metaspades” function of SPAdes [66,67]. Viral contigs were selected using VirSorter2 (version 2.2.4, only including dsDNA phages with a minimum length of 2,000 bp) [76] and CheckV (version 1.0.1, database v1.5) [77]. Contigs encoding less than two genes or with no viral gene detected were discarded for further analyses. The selected contigs were aligned with available bacterial genomes of the strains included in the bacterial community using BLAST [78]. Those with a similarity up to 99% in at least 99% of their length, with an *E*-value = 0.0 were considered positive hits. Hits were confirmed by read mapping against corresponding bacterial strains and visualized with IGV. Prophage activation was assumed if reads were uniquely mapped in a specific region. Bacteria with positive hits were searched for prophages in their genomes using PHASTEST [79], and viral contigs that hit with each bacterium were compared using BLAST [78] with prophages detected to confirm their proviral origin.

### Analysis of variability and selection in the evolved phages

Genomes of the evolved phage variants obtained from single plaque isolation were classified and reordered based on the reassembly of their parents. Functional annotation of parents and variants was carried out using Pharokka (v1.4.1) (S4A Data) [64]. Genome alignment was performed with MAFFT (version 7.520) [80] to identify variable positions. To evaluate selection in mutated genes, we calculated the dN/dS ratio with the BUSTED [31]. To do it for every position of the protein, we calculated the differential between the rate of non-synonymous substitutions (*β*) and the rate of synonymous substitutions (*ɑ*) and the posterior probability of one being higher than the other with the FUBAR test; A Fast, Unconstrained Bayesian AppRoximation for Inferring Selection [32]. Selection tests were performed using Datamonkey [81,82].

When evolved phages were recombinants of two ancestral phages, recombination events were detected using an automated query versus reference analysis in RDP4 for the three lines separately [83]. Recombination events were considered when confirmed by at least 5 of the 7 utilized methods: RDP, GENECONV, MaxChi, BootScan, Chimaera, 3Seq, and SiScan. When one of the parental sequences of the recombinant was unknown, recombination was detected by an in-depth observational analysis of the MAFFT [80] alignment using MEGAX [74] of the recombinant with the known parental and other sequences of similar phages available in the National Center for Biotechnology Information (NCBI) database, as well as genome visualization with gggenomes [75].

Amino acid sequences of RBPs and the central tail fiber J (if present) were aligned using MAFFT [80] to find variable positions in the amino acid sequence. Protein domains were identified when possible using InterProScan [34] or by visual observation based on the 3D trimeric structure of the protein, predicted with AlphaFold Server, which uses AlphaFold3 [33]. Coloring regions of interest and variable positions of the protein sequence alignment representation as “spheres” was done with Pymol [84]. HHpred [85] was used to assess similarity to other protein groups.

## Supporting information

S1 Fig Evolved variants K74_evo1 and K74_evo4 of phage B and differences in the increment of titer over time. S1A. Representation of the homology of phage variants (K74_evo1 and K74_evo4) and the ancestor (Phage B—K74PH129C2) using the R package gggenomes [75]. The phages are represented as the annotated coding sequences (CDSs). Functions are represented by different colors specified in the legend. The function of deleted fragments with known functions is indicated with *: *1. SAM-dependent methyltransferase. *2. dGTPase inhibitor. S1B. Graphic representation of the difference in the increment of titer per time for each phage. Calculated as follows: ∆Log_10_(titer) = Log_10_(Ti titer)–Log_10_(Tf titer), being Ti = initial time and Tf = final time. The increment of titer was calculated in 6 hours. The data underlying this Figure can be found in S5 Data.
(TIFF)

S2 Fig Variable position frequency comparison between variant calling and phage variant isolation and evaluation of effects of mutation patterns on the efficacy of plating (EOP). S2A. Comparison of the allele frequency of each mutation detected through variant calling in the phage community and the variants isolated in each line/passage combination. The data underlying this Figure can be found in S4B and S4C Data tabs in S4 Data file. S2B. Analysis of EOP modifications of phage variants compared to their ancestral phage in a subset of strains. Variation in EOP is represented on a logarithmic scale. EOPs lower than the ancestor are represented in shades of purple and higher in shades of green. The data underlying this Figure can be found in S8 Data.
(TIFF)

S1 Table 77 *Klebsiella* reference strains collection. ENA: European Nucleotide Archive.
(XLSX)

S2 Table Description of the isolated evolved phages. They are named according to their ancestral phage. Isolation lineage, passage, and strain, ancestral phages, and genome length of each evolved phage are given in the table. Evo: evolved, non-recombinants. Rec: recombinants. Bp: base pairs.
(XLSX)

S3 Table Comparison of Poisson rates test comparing mutations per position in proteins with observed accumulation of mutations with mutations per position in the rest of the genome. To conduct, we considered mutations affecting different positions found in passages 40 and/or 69 in each lineage separately. RBP: receptor-binding protein, STK: serine-threonine kinase, VC: variant calling, WGA: whole genome alignment, ROTG: rest of the genome.
(XLSX)

S4 Table Mutations in the whole genomes, amino acid changes in the RBP, and EOP differences of phage variants compared to the ancestral phage. CDS: coding sequence, FUBAR: A Fast, Unconstrained Bayesian AppRoximation for Inferring Selection, BF: Bayes factor, AF: Allele frequency, RBP: Receptor-binding protein, INS: insertion, bp: base pair, aa: amino acid, EOP: efficacy of plating.
(XLSX)

S5 Table Detection of putative double-stranded DNA potential prophages in the phage cocktail evolution experiment. Bp: base pairs, NCBI: National Center for Biotechnology Information.
(XLSX)

S1 Data Average sequencing depth of the genome sequences of the phages included in the community at different passages and lines. A correction was applied for pairs of closely related phages at depth ratios below 20/80 (Phages B and C). Mutations detected at passage 0 in the read mapping of each phage at a consistent specific ratio corresponded to discrepancies between the phages. We calculate the relative proportion of mapped reads corresponding to each phage by calculating the average of the frequency at which each mutation appears. We multiply this value (from 0 to 1) by the total number of reads mapped. We cannot apply this correction for pairs of close relative phages where the ratio is close to 50−50 (Phages I and J, and G and H. These phages are represented together).
(XLSX)

S2 Data Phylogenetic relationship and infection pattern against the 77 *Klebsiella* reference serotypes of the phages in the community. S2A. Genetic distance matrix of the phages. S2B. Infection matrix of the phages against the 77 *Klebsiella* reference serotypes collection. Data correspond to the matrix presented in Ferriol-González and colleagues 2024 [23], including doubtful or inconsistent interactions for 10 of the 12 phages. S2C. Revised host range of phage F at a titer of 10^8^ PFU/mL by serial dilution spot test in a subset of strains of interest. To perform the consensus of the replicates, if plaques were observed in every one of them at any dilution, the phage-bacteria interaction was scored with “2”. If the result was inconsistent or single plaques were not observed in the spots of some replicates, the interaction was scored with “1”. S2D. Revised host range of phage A at a titer of 10^8^ PFU/mL by serial dilution spot test in a subset of strains of interest. Criteria for obtaining consensus results were the same as those described in S2C.
(XLSX)

S3 Data Host range of the three evolution lines of the phage community at different passages over the 77 *Klebsiella* reference serotypes collection. The host range was assessed by serial dilution spot test. R: replicate. Two replicates were performed, and a third one was performed for inconsistent results. S3A. Host range of the initial phage community. S3B. Host range of the three evolution lines at passage 20. S3C. Host range of the three evolution lines at passage 40. S3D. Host range of the three evolution lines at passage 69.
(XLSX)

S4 Data Annotation of the phages in the community and analysis of mutations detected during the evolution experiment. CDS: coding sequence, RBP: receptor-binding protein. S4A. Annotation of the ancestral phages of the initial phage community. S4B. Variant calling analyses of the evolved phage communities. S4C. Variable positions of the whole genome alignment of parental and evolved phages. -: deletion. S4D. Results of the Branch-Site Unrestricted Statistical Test for Episodic Diversification (BUSTED) for CDSs where the tested dN/dS ratio was > 1. dN/dS: ratio of non-synonymous to synonymous substitutions, CoV: Coefficient of variation.
(XLSX)

S5 Data Differences in the increment of titer over time between phage B and evolved variants K74_evo1 and K74_evo4. Raw data, ANOVA results, and Post Hoc Tukey results.
(XLSX)

S6 Data FUBAR test (Fast, Unconstrained Bayesian AppRoximation for Inferring Selection) results. Graphic representation of the *β*–α value for each position of the CDS, with dN/dS > 1 indicating exact positions under positive selection. Bayes factor for the prediction of positive selection on positions with posterior probabilities of [*α* < *β*] < 0.9% were > 10 (strong evidence). aa: amino acid. S6A. Positions per protein under the effect of selection. S6B. *β*–*α* value for each position.
(XLSX)

S7 Data Protein sequence alignment of mutated tail proteins potentially involved in host recognition or attachment. RBP: receptor-binding protein, aa: amino acid.
(XLSX)

S8 Data Efficacy of plating (EOP) variation between phage variants of phages F and A compared to their ancestral phage in a subset of strains. S8A. Data of phage F and variants. S8B. Data of phage A and variants.
(XLSX)

## Abbreviations

BUSTED: Branch-Site Unrestricted Statistical Test for Episodic Diversification
CDS: coding sequence
CFU: colony-forming unit
dsDNA: double-stranded DNA
EOP: efficacy of plating
FUBAR: Fast, Unconstrained Bayesian AppRoximation for Inferring Selection
IGV: Integrative Genomics Viewer
PFU: plaque-forming unit
RBP: receptor-binding protein
