# Supplementary figures and images for: The host range of generalist and specialist phages in capsule-diverse Klebsiella hosts is driven by the evolvability of receptor-binding proteins
Source: PLoS Biol. 2025 Nov 26;23(11):e3003515. doi: 10.1371/journal.pbio.3003515 (PMC12654880; doi:10.1371/journal.pbio.3003515)

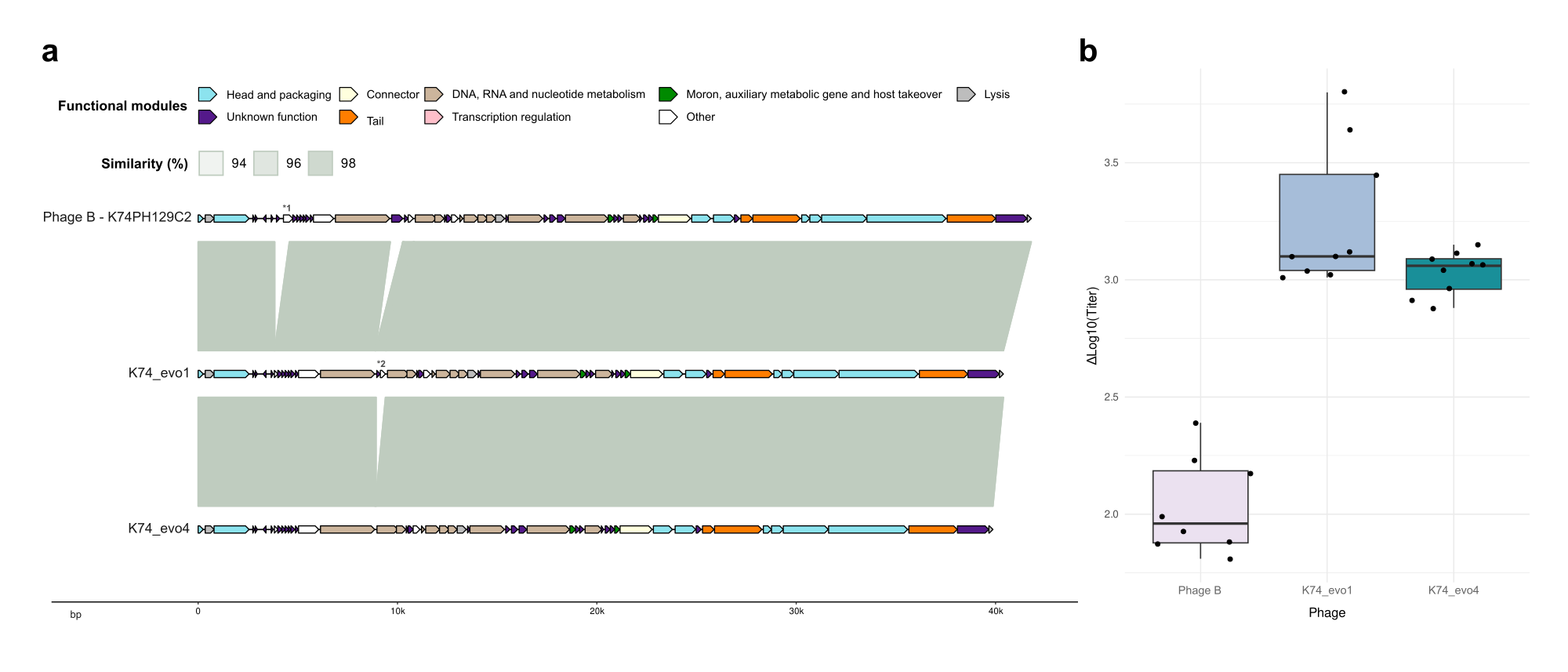

Supplement: S1 Fig — S1A. Representation of the homology of phage variants (K74_evo1 and K74_evo4) and the ancestor (Phage B—K74PH129C2) using the R package gggenomes [75]. The phages are represented as the annotated coding sequences (CDSs). Functions are represented by different colors specified in the legend. The function of deleted fragments with known functions is indicated with *: *1. SAM-dependent methyltransferase. *2. dGTPase inhibitor. S1B. Graphic representation of the difference in the increment of titer per time for each phage. Calculated as follows: ∆Log10(titer) = Log10(Ti titer)–Log10(Tf titer), being Ti = initial time and Tf = final time. The increment of titer was calculated in 6 hours. The data underlying this Figure can be found in S5 Data. (TIFF) [file pbio.3003515.s001.tiff]

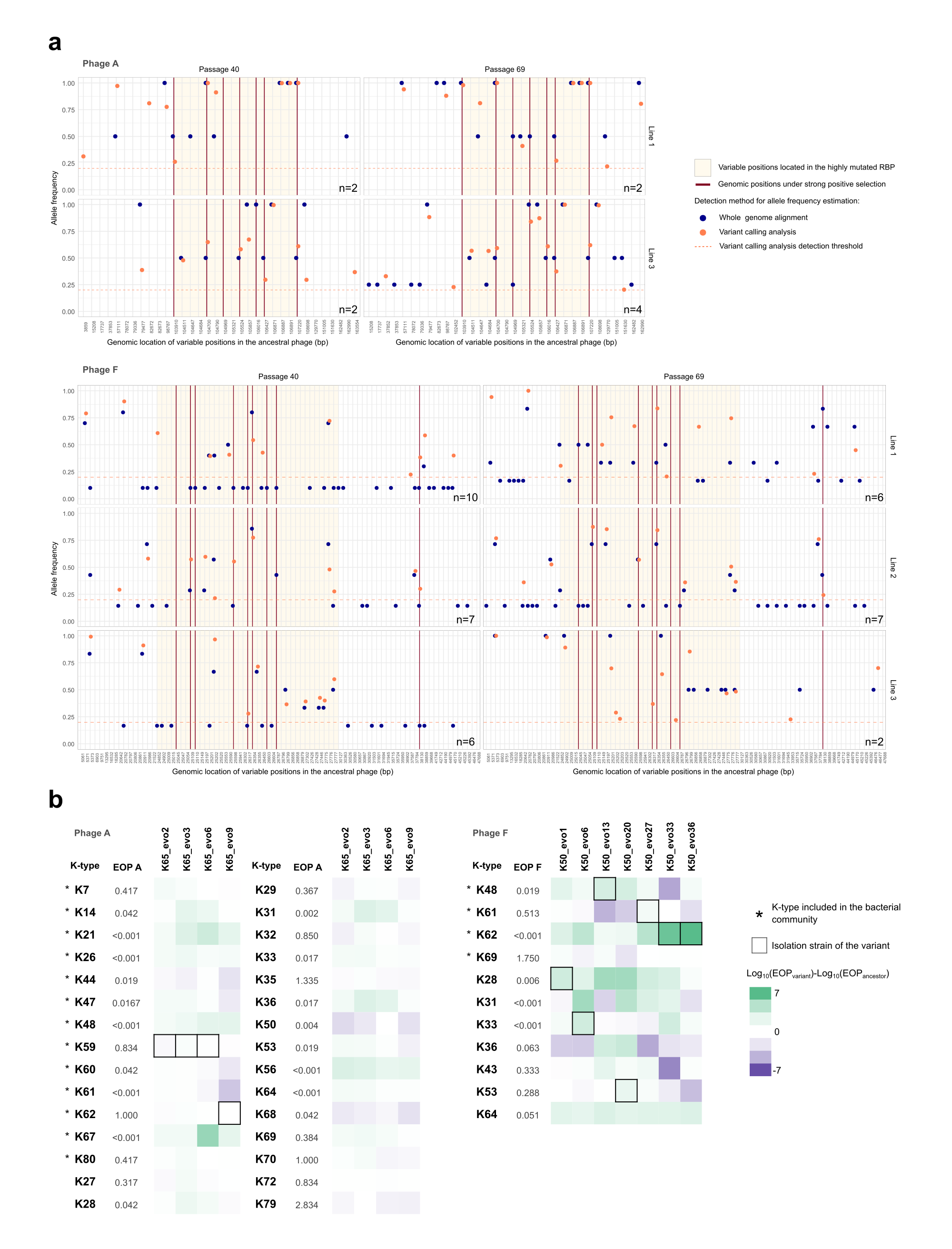

Supplement: S2 Fig — S2A. Comparison of the allele frequency of each mutation detected through variant calling in the phage community and the variants isolated in each line/passage combination. The data underlying this Figure can be found in S4B and S4C Data tabs in S4 Data file. S2B. Analysis of EOP modifications of phage variants compared to their ancestral phage in a subset of strains. Variation in EOP is represented on a logarithmic scale. EOPs lower than the ancestor are represented in shades of purple and higher in shades of green. The data underlying this Figure can be found in S8 Data. (TIFF) [file pbio.3003515.s002.tiff]
